# Influences and Practices in Colorectal Cancer Screening Among Health Care Providers Serving Northern Plains American Indians, 2011–2012

**DOI:** 10.5888/pcd13.160267

**Published:** 2016-12-15

**Authors:** Melanie Nadeau, Anne Walaszek, David G. Perdue, Kristine L. Rhodes, Donald Haverkamp, Jean Forster

**Affiliations:** 1 North Dakota State University, Department of Public Health, Fargo, North Dakota; 2 American Indian Cancer Foundation, Minneapolis, Minnesota; 3 Minnesota Gastroenterology PA, Minneapolis, Minnesota; 4 Centers for Disease Control and Prevention, Division of Cancer Prevention and Control, Albuquerque, New Mexico; 5 University of Minnesota, Minneapolis, Minnesota.

## Abstract

**Introduction:**

The epidemiology of colorectal cancer, including incidence, mortality, age of onset, stage of diagnosis, and screening, varies regionally among American Indians. The objective of the Improving Northern Plains American Indian Colorectal Cancer Screening study was to improve understanding of colorectal cancer screening among health care providers serving Northern Plains American Indians.

**Methods:**

Data were collected, in person, from a sample of 145 health care providers at 27 health clinics across the Northern Plains from May 2011 through September 2012. Participants completed a 32-question, self-administered assessment designed to assess provider practices, screening perceptions, and knowledge.

**Results:**

The proportion of providers who ordered or performed at least 1 colorectal cancer screening test for an asymptomatic, average-risk patient in the previous month was 95.9% (139 of 145). Of these 139 providers, 97.1% ordered colonoscopies, 12.9% ordered flexible sigmoidoscopies, 73.4% ordered 3-card, guaiac-based, fecal occult blood tests, and 21.6% ordered fecal immunochemical tests. Nearly two-thirds (64.7%) reported performing in-office guaiac-based fecal occult blood tests using digital rectal examination specimens. Providers who reported receiving a formal update on colorectal cancer screening during the previous 24 months were more likely to screen using digital rectal exam specimens than providers who had received a formal update on colorectal cancer screening more than 24 months prior (73.9% vs 56.9%, respectively, χ^2^ = 4.29, *P* = .04).

**Conclusion:**

Despite recommendations cautioning against the use of digital rectal examination specimens for colorectal cancer screening, the practice is common among providers serving Northern Plains American Indian populations. Accurate up-to-date, ongoing education for patients, the community, and health care providers is needed.

## Introduction

In 2012, more than 134,000 people were diagnosed with colorectal cancer (CRC) in the United States (1). Approximately 70% to 75% of those diagnosed with CRC are considered to be at average risk for the disease (2). Screening people at average risk decreases CRC incidence and mortality (2,3). Screening and reduction of identifiable risk factors have contributed to long-term declines in CRC incidence since the mid-1980s (4).

CRC incidence, mortality, age of onset, and stage of diagnosis vary regionally among American Indians and Alaska Natives (AI/ANs), with higher rates of CRC in the Alaska, Northern Plains, and Southern Plains regions (5). One study showed that AI/ANs were younger at age of diagnosis and more likely to be diagnosed with CRC at an advanced stage than whites (5). AI/ANs are less likely than whites to be up to date with CRC screening (6–9). According to data from the 2014 Government Performance and Results Act, 37.5% of AI/ANs nationally were up to date with CRC screening (10). By comparison, in 2013, 58.2% of the general population of the United States was up to date with recommended CRC screening, based on data from the National Health Interview Survey (11).

Provider recommendation is an important determinant of whether or not a patient undergoes CRC screening (12,13). Providers need to be aware of the most current CRC screening recommendations (14). One challenge to screening that remains among primary care physicians is the practice of screening for occult fecal blood using specimens obtained from digital rectal examinations (DREs), even though this practice detects fewer than 5% of patients with CRC or advanced adenomas (15,16). A web-based survey suggested that screening by using DRE stool specimens is still pervasive among Indian Health Service providers (17). A better understanding of factors that influence provider screening recommendations is needed to inform programs that encourage guideline-based screening practices. The Improving Northern Plains American Indian Colorectal Cancer Screening (INPACS) study was undertaken to improve understanding of CRC screening among health care providers serving Northern Plains American Indians.

## Methods

The INPACS project was created to learn about CRC screening at clinics serving American Indians across the Northern Plains and to then use project data to guide the development of clinical strategies for improving screening practices and capacity. The American Indian Cancer Foundation invited the 54 Northern Plains facilities that provide health care services primarily to American Indians to participate in the INPACS project. These facilities included a combination of Indian Health Service (IHS), tribal, and urban American Indian health systems in the following states: Minnesota, Montana, Nebraska, North Dakota, South Dakota, Wisconsin, and Wyoming. All INPACS materials and instruments were reviewed and approved by the National Institutional Review Board (IRB) at Indian Health Service headquarters, the IHS Great Plains Area IRB, and the Rocky Mountain Tribal IRB of the Montana–Wyoming Tribal Leaders Council.

### Sample and data collection

Data were collected in person by INPACS staff members from a sample of 145 health care providers from 27 of the 54 health clinics who agreed to participate. Data were collected from May 2011 through September 2012. The 90-minute INPACS program, which included data collection through a self-administered provider assessment followed by a continuing medical education course, was scheduled during a typical clinic staff meeting to ensure high attendance. Attendees learned details of the INPACS program through a verbal presentation and reading the informed consent document. The purpose of the INPACS continuing medical education course was twofold: first, to offer an incentive to providers to compensate for their time away from clinic hours and second, to support the clinic in being up to date on CRC screening practices by their participation in the program. Eligible participants were primary care providers who served American Indian adults: physicians, nurse practitioners, physician assistants, and clinical nurse-midwives who treated adult patients. The self-administered assessment administered to providers did not include individual identifiers, and clinic-specific data were confidential.

### Self-administered provider assessment and variables

After informed consent was obtained, participants completed a self-administered provider assessment under supervision of the INPACS staff. This assessment was designed to assess provider screening practices, perceptions, and knowledge. The National Cancer Institute’s *National Survey of Primary Care Physicians’ Cancer Screening Recommendations and Practice for Breast, Cervical, Colorectal, and Lung Cancer Screening* was used to guide the development of the self-administered provider assessment, which included 32 questions (18). To assess the types of CRC screenings that providers ordered or performed for their patients, participants were asked the following question: “During a typical month, how many times do you order or perform the following screening tests for your asymptomatic, average-risk patients: 1) guaiac of digital rectal exam (DRE) specimen, 2) take home 3-card fecal occult blood tests (FOBT), 3) fecal immunochemistry test (FIT), 4) screening flexible sigmoidoscopy, 5) screening colonoscopy, 6) other (specify).”

To discern variables that might influence CRC screening recommendations, participants were asked the following: 1) “How many years have you been in practice since finishing your training,” 2) “When was the last time you received a formal update (CME course or in-service) on CRC screening,” and 3) “To what extent do the following influence your recommendation for CRC screening for your patients: a) US Preventive Services Task Force recommendations, b) American Cancer Society/multi-society guidelines [guidelines issued by the American Cancer Society, the US Multi-Society Task Force on Colorectal Cancer, and The American College of Radiology], c) whether the patient has third-party insurance, including Medicare and Medicaid, d) availability of screening tests (other than FOBT), e) how others in my practice or local community provide CRC screening for their patients, f) my patients’ preferences for CRC screening, and g) the availability of IHS or tribal funds for screening.” We used χ^2^ tests to examine differences in categorical data and considered significance at *P* < .05. Analyses were conducted using Epi Info 7 (19).

We hypothesized that providers who began practice before 2008 would be more likely to order or perform in-office FOBTs using a digital rectal exam (DRE) specimen for screening than providers who started practicing in 2008 or later. The year 2008 was used as the cutoff because that was the year in which updated CRC screening recommendations were released by the US Preventive Services Task Force and a joint guideline was issued by the American Cancer Society, the US Multi-Society Task Force on Colorectal Cancer, and The American College of Radiology (14). We also hypothesized that providers who had received a formal update on CRC screening during the previous 24 months would be less likely to order or perform FOBT by using a DRE specimen as a screening test than providers who had received a formal update on CRC screening more than 24 months ago.

## Results

The proportion of providers who ordered or performed at least one CRC screening test for an asymptomatic, average-risk patient in the previous month was 95.9% (139 of 145). Of the 139 providers, 97.1% (n = 135) ordered screening colonoscopies, 12.9% (n = 18) ordered screening flexible sigmoidoscopies, 73.4% (n = 102) ordered take-home 3-card FOBT, 21.6% (n = 30) ordered fecal immunochemical tests, and 64.7% (n = 90) ordered or performed office-based FOBT using DRE specimens. The number of times per month any given provider ordered or performed a particular CRC screening test for their asymptomatic, average-risk patients is presented in Table 1.

**Table 1:** Number of Times Health Care Providers (N = 139) Ordered or Performed a Colorectal Screening Test for Asymptomatic, Average-Risk Patients During a Typical Month, Northern Plains^a^ American Indians, May 2011–September 2012

Test	No. (%) of Health Care Providers, by Times per Month Test Performed or Ordered				
	0 Times	1–10 Times	11–20 Times	21–40 Times	>40 Times
**Guaiac of digital rectal examination (DRE) specimen**	49 (35.3)	85 (61.2)	2 (1.4)	2 (1.4)	1 (0.7)
**Take** **home 3-card fecal occult blood test**	37 (26.6)	80 (57.6)	17 (12.2)	4 (2.9)	1 (0.7)
**Fecal immunochemistry test**	109 (78.4)	20 (14.4)	2 (1.4)	5 (3.6)	3 (2.2)
**Screening flexible sigmoidoscopy**	121 (87.1)	16 (11.5)	1 (0.7)	1 (0.7)	0 (0.0)
**Screening colonoscopy**	4 (2.9)	112 (80.6)	15 (10.8)	6 (4.3)	2 (1.4)

a Includes Minnesota, Montana, Nebraska, North Dakota, South Dakota, Wisconsin, and Wyoming. Data are from the Improving Northern Plains American Indian Colorectal Cancer Screening project.

A similar proportion of providers who began practice in 2008 or later reported ordering or performing an office-based FOBT using a DRE specimen compared with those who began practice before 2008 (69.6% vs 63.8%, respectively; χ^2^ = 0.28, *P* = .60). Providers who reported receiving a formal update on CRC screening during the previous 24 months were more likely to perform a FOBT using DRE specimens than providers who had received a formal update on CRC screening more than 24 months prior (73.9% vs 56.9%, respectively; χ^2^ = 4.29, *P* = .04).

When stratifying by type of provider, mid-level providers (nurse practitioners, physician assistants, certified nurse-midwives) were more likely than physicians to have begun practice in 2008 or later (24.6% vs 9.5%, respectively, χ^2^ = 5.76, *P* = .02) (Table 2). However, mid-level providers were less likely to have received a formal update on CRC screening during the previous 24 months than physicians (36.9% vs 58.1%, respectively, χ^2^ = 6.22, *P* = .01). A similar proportion of mid-level providers ordered or performed an office-based FOBT using a DRE specimen compared with physicians (63.1% vs 66.2%, respectively; χ^2^ = 0.15 *P* = .70) (Table 2). We also found no particular method of CRC screening that mid-level providers recommended more than physicians.

**Table 2:** Influences and Practices of Physicians and Mid-Level Health Care Providers (N = 139) Serving Northern Plains^a^ American Indians Regarding Colorectal Cancer Screening, by Type of Provider, May 2011–September 2012

Characteristic	Type of Health Care Provider		
	MD/DO (n = 74), n (%)	NP/PA/CNM (n = 65), n (%)	*P* Value^b^
**Began clinical practice 2008 or later**	7 (9.5)	16 (24.6)	.02
**≤24 Months since last CRC training**	41 (55.4)	24 (36.9)	.01
**Screening tests recommended**			
**Screening colonoscopy**	70 (94.6)	65 (100.0)	.06
**Screening flexible sigmoidoscopy**	12 (16.2)	6 (9.2)	.22
**Take home 3-card fecal occult blood test**	51 (68.9)	51 (78.5)	.21
**Fecal immunochemistry test**	18 (24.3)	12 (18.5)	.40
**Guaiac of digital rectal examination specimen**	49 (66.2)	41 (63.1)	.70

Abbreviations: CNM, certified nurse-midwife; CRC, colorectal cancer; DO, doctor of osteopathy; MD, medical doctor; NP, nurse practitioners; PA, physician assistant.

a Includes Minnesota, Montana, Nebraska, North Dakota, South Dakota, Wisconsin, and Wyoming. Data are from the Improving Northern Plains American Indian Colorectal Cancer Screening project.

b Determined by χ^2^ test.

Most providers reported that US Preventive Services Task Force recommendations were very influential or somewhat influential in their screening recommendations (98.5%) followed by American Cancer Society/Multi-Society guidelines (94.1%) (Table 3). The patient’s preference for the type of test was also influential for most providers (89.7%), although 30.2% of providers reported that patients frequently preferred the provider to choose the appropriate test for them. The availability of IHS or tribal funds for screening influenced the tests ordered or performed by 69.6% of providers. Finally, third party medical coverage (private insurance, Medicare and Medicaid) was reported by 40.4% of providers to influence their CRC screening decisions. The influence various screening guidelines and other factors had on screening recommendations did not vary significantly by provider type (Table 3).

**Table 3:** Factors Influencing Health Care Providers’ (N = 139) Colorectal Cancer Screening Practices for Northern Plains^a^ American Indians, by Level of Influence and Type of Provider, May 2011–September 2012

Influence	No. of Providers Who Responded	Very Influential	Somewhat Influential	Not Influential	*P* Value^b^
		n (%)			
**US Preventive Services Task Force recommendations**	133	96 (72.2)	35 (26.3)	2 (1.5)	—
**MD/DO**	72	49 (68.1)	22 (30.6)	1 (1.4)	.48
**NP/PA/CNM**	61	47 (77.1)	13 (21.3)	1 (1.6)	
**American Cancer Society/multi-society guidelines^c^**	135	93 (68.9)	34 (25.2)	8 (5.9)	—
**MD/DO**	73	49 (67.1)	20 (27.4)	4 (5.5)	.80
**NP/PA/CNM**	62	44 (71.0)	14 (22.6)	4 (6.5)	
**Whether the patient has third party insurance, including Medicare and Medicaid**	136	18 (13.2)	37 (27.2)	81 (59.6)	—
**MD/DO**	73	10 (13.7)	21 (28.8)	42 (57.5)	.87
**NP/PA/CNM**	63	8 (12.7)	16 (25.4)	39 (61.9)	
**Availability of screening tests (other than FOBT)**	135	43 (31.9)	58 (43.0)	34 (25.2)	—
**MD/DO**	72	27 (37.5)	31 (43.1)	14 (19.4)	.17
**NP/PA/CNM**	63	16 (25.4)	27 (42.9)	20 (31.8)	
**How others in my practice or local community provide CRC screening for their patients**	135	13 (9.6)	56 (41.5)	66 (48.9)	—
**MD/DO**	72	7 (9.7)	31 (43.1)	34 (47.2)	.91
**NP/PA/CNM**	63	6 (9.5)	25 (39.7)	32 (50.8)	
**My patients’ preferences for CRC screening (n = 135)**	135	36 (26.7)	85 (63.0)	14 (10.4)	
**MD/DO**	73	17 (23.3)	47 (64.4)	9 (12.3)	.52
**NP/PA/CNM**	62	19 (30.7)	38 (61.3)	5 (8.1)	
**Availability of IHS or tribal funds for screening**	135	47 (34.8)	47 (34.8)	41 (30.4)	—
**MD/DO**	73	26 (35.6)	23 (31.5)	24 (32.9)	.65
**NP/PA/CNM**	62	21 (33.9)	24 (38.7)	17 (27.4)	

Abbreviations: CNM, certified nurse-midwife; CRC, colorectal cancer; DO, doctor of osteopathy; DRE, digital rectal exam; IHS, Indian Health Service; MD, medical doctor; NP, nurse practitioners; PA, physician’s assistant; —, not applicable.

a Includes Minnesota, Montana, Nebraska, North Dakota, South Dakota, Wisconsin, and Wyoming. Data are from health care providers surveyed for the Improving Northern Plains American Indian Colorectal Cancer Screening project.

b Difference between level of influence and type of provider determined by χ^2^ test.

c Guidelines issued by the American Cancer Society, the US Multi-Society Task Force on Colorectal Cancer, and The American College of Radiology (14).

## Discussion

Provider adherence to evidence-based screening practices is necessary for CRC prevention and control. This study focused on the CRC screening practices of 145 practitioners who served at American Indian health facilities in the Northern Plains. Although most providers in our sample reported offering CRC screening, adherence to guidelines on recommended screening practices could be improved. Sixty-five percent of our sample used DRE stool specimens for in-office FOBTs among their CRC screening options. These findings are consistent with the findings of a previous survey of IHS and tribal health providers in which 23.0% of providers who screened with FOBT recommended only in-office FOBT specimens (17) and a survey of providers nationwide that showed that 32.5% used in-office FOBT as their only method of CRC screening (20). The use of DRE stool specimens for FOBT did not vary significantly by the length of time providers had been in practice. Counterintuitively, providers who reported receiving education on CRC screening recommendations in the previous 24 months were more likely to report using DRE stool specimens to complete an in-office FOBT.

The guaiac-based testing of DRE samples was recommended by most medical societies as a CRC screening test in the 1980s and 1990s (21,22) and as such was commonly taught in medical training programs. However, data from a study published in 2005 found the practice to have a sensitivity for advanced neoplasia (cancer or advanced adenomatous polyps) of only 4.9% (15). Concerns that such testing may give patients and practitioners false assurance about cancer status and dissuade more appropriate screening led professional societies in the mid-1990s to urge abandonment of the practice (23).

Although this study centered on self-administered provider assessments, focus groups conducted with providers in another component of the INPACS program found suboptimal awareness that the use of a single stool sample on a FOBT card after a DRE was no longer a recommended screening test. Many factors emerged during the focus groups that may help explain the continuation of this practice. Among these, focus group participants often cited their low expectations for patients completing other forms of screening. Pressure to increase screening rates to meet targets set by the Government Performance Results Act also played a role for some. Finally, many participants reported being told by health administrators to “at least do a DRE test.” The finding that study participants who reported having received continuing medical education or some other update on CRC screening in the previous 24 months were more likely to conduct DRE screening suggests that those tasked with conducting provider education may not be communicating clearly that these practices are no longer recommended. Strategies that may help to discourage DRE in practice include development of a clinic policy of recommended screenings and frequent continuing medical education on updated CRC screening.

Although national recommendations were the most commonly cited influence on screening practices reported by providers, patient preference and socioeconomic factors were also cited. For almost half of the providers (40.4%) a recommendation for CRC screening was influenced by the availability of health insurance coverage. Moreover, the availability of IHS or tribal funds for screening influenced the majority (69.6%) of providers’ CRC screening recommendations. Funding restrictions may also be affecting the community’s ability to pursue new screening options. This may explain why DRE and take home 3-card FOBT and are being performed more frequently than FIT.

Access to CRC screening for American Indians can be particularly challenging. Health care coverage is complex, consisting of a mixture of IHS, tribal, public, and commercial payers. According to the Institute of Medicine, IHS is underfunded and unable to meet the needs of the population it serves (24). One possible result of underfunding may be rationing of noncritical services such as screening colonoscopy. Tribal health systems vary widely in the resources available them, often being primarily influenced by the success of tribal gaming and other business enterprises. Factors such as tribal enrollment, tribal employment, and county of residence may all affect the health services available to American Indians.

The Affordable Care Act could provide American Indians with more choices that could lead to more adequate health insurance coverage. Depending on eligibility and the coverage available, American Indians can continue to use IHS, tribal, or urban Indian health programs, enroll in a qualified health plan through the Health Insurance Marketplace, or access coverage through Medicare or Medicaid (25). Whether the further removal of financial barriers to CRC screening will affect screening uptake remains to be seen.

In addition to cost, many other barriers to endoscopic screening (colonoscopy, sigmoidoscopy) for CRC may influence provider recommendations. Many tribal health systems are located far from endoscopy services that can provide screening or follow-up procedures. In addition to travel, other barriers are low levels of health literacy about screening benefits, fear of embarrassment or findings, traditional health beliefs, and mistrust of medical practices that do not serve primarily American Indians (26–28). A solution that may improve screening capacity may be to fully implement FIT testing with an adequate tracking and follow-up system and to keep colonoscopy referrals to a minimum by reserving colonoscopy for those with positive FIT results.

The American College of Physicians guidelines reinforce the importance of shared decision making between provider and patient (29). Shared decision making involves the provider discussing the pros and cons of CRC screening options with the patient and then assisting the patient in selecting the most appropriate test the patient is willing and able to complete. Despite apparent efforts to share decision making, many providers from our survey (30.2%) reported their patients often wanted them to choose a test for them. Work to validate this impression from the patient’s perspective is needed.

A limitation of the INPACS study is that the provider assessment was self-administered and allowed for over- or under-reporting of provider behaviors on CRC screening recommendations and practices. Another limitation of the study is the low participation rate. Although the study team visited 40 of the 54 health clinics serving American Indians in the Northern Plains, the 145 survey participants were the health care providers from 27 clinics that participated in both components of the study (facility assessment and provider assessment). The results of this study are also limited in their generalizability because they are specific to American Indian clinics in the Northern Plains.

Disparities in CRC incidence, mortality, and screening among Northern Plains American Indians emphasize the need to better understand the barriers this population faces in CRC prevention and control. DRE use is often based on the perception that it may be the only opportunity to screen the patient, even though it is not a best practice and may lead to incorrect results. The continued use of this test points out the critical need to deliver up-to-date, ongoing provider and patient education in American Indian health systems to ensure this population’s health providers practice in concordance with national CRC screening recommendations. The Centers for Disease Control and Prevention offers free online continuing medical education on CRC screening for providers (30). After completing the self-administered provider assessment for this study, all providers participated in a continuing medical education session that reviewed current CRC screening recommendations that included data on why screening using office-based FOBT with DRE sample is discouraged. Educating patients and the community is equally important. Accurate, up-to-date, ongoing education for patients, the community, and health care providers is needed. Resolving the issue of up-to-date provider screening recommendations is a critical step, but much work will be needed to address the economic and sociocultural barriers that hinder effective CRC prevention and control in this population.
